# Trial of the Boston Apothecary

**Published:** 1850-11

**Authors:** 


*Trial of the Boston Apothecary.*—The Boston apothecary, Wakefield, who dispensed ten grains of corrosive sublimate for calomel, has been tried for manslaughter, and acquitted. It appeared on evidence, that the poison was probably not the cause of death.

